# Detecting Lateral Motion using Light’s Orbital Angular Momentum

**DOI:** 10.1038/srep15422

**Published:** 2015-10-23

**Authors:** Neda Cvijetic, Giovanni Milione, Ezra Ip, Ting Wang

**Affiliations:** 1 Optical Networking and Sensing Department, NEC Laboratories America, Princeton, NJ, 08540 USA

## Abstract

Interrogating an object with a light beam and analyzing the scattered light can reveal kinematic information about the object, which is vital for applications ranging from autonomous vehicles to gesture recognition and virtual reality. We show that by analyzing the change in the orbital angular momentum (OAM) of a tilted light beam eclipsed by a moving object, lateral motion of the object can be detected in an arbitrary direction using a single light beam and without object image reconstruction. We observe OAM spectral asymmetry that corresponds to the lateral motion direction along an arbitrary axis perpendicular to the plane containing the light beam and OAM measurement axes. These findings extend OAM-based remote sensing to detection of non-rotational qualities of objects and may also have extensions to other electromagnetic wave regimes, including radio and sound.

Remote sensing using light^1^,^2^ has predominantly exploited light’s temporal and frequency degrees of freedom. Light’s orbital angular momentum (OAM)^3^ represents another fundamental optical degree of freedom^4^: space. In the paraxial approximation, a light beam can have a helical phase structure described by exp(*ilϕ*),where *l* = 0, ±1, ±2, …, and *ϕ* denotes the azimuthal coordinate^3^. Such a beam carries an orbital angular momentum (OAM) of *lħ* per photon along its beam axis^3^, *ħ* denoting Planck’s constant *h* divided by *2π*. Light’s OAM has been studied in a plurality of contexts including optical manipulation and trapping, quantum information processing, imaging, astronomy, high-speed transmission, and remote sensing of rotational Doppler frequency shifts and rotational structure symmetries in objects^4^,^5^,^6^,^7^,^8^,^9^,^10^,^11^,^12^,^13^,^14^,^15^,^16^,^17^. We show experimentally that by analyzing the change in OAM of a tilted light beam eclipsed by a moving object, a non-rotational quality of the object – its lateral motion – can be detected using a single light beam. These results can be applied in autonomous vehicles, gesture recognition, and virtual reality systems to achieve lateral motion detection without object image reconstruction. Moreover, since OAM is a general electromagnetic wave phenomenon, the work may also have extensions to other wave regimes, including radio and sound, and could potentially be applied to lateral motion detection in microscopy and astronomy.

Light’s OAM can be intrinsic or extrinsic^18^. Intrinsic OAM, described by the azimuthal phase factors exp(*ilϕ*) referred to as OAM basis states, is defined with respect to the beam axis, typically taken to be the **z** axis of the cylindrical polar coordinate system. However, when defined with respect to a different, so-called measurement axis, **n**, extrinsic OAM may be observed^18^. Extrinsic OAM is associated with changes in the beam’s center of gravity (i.e. centroid of intensity^19^), which in the paraxial approximation is a function of several parameters, including beam tilt (angular misalignment between **z** and **n**), obstruction, profile, and waist size. Consequently, when a light beam is tilted and/or obstructed, its OAM spectrum— the optical power in each OAM basis state — can change due to extrinsic OAM^7^,^20^,^21^,^22^.

For simultaneous beam tilt and lateral misalignment, OAM spectral asymmetry about the launch OAM state can arise^21^,^22^,^23^. We experimentally show that in the case of simultaneous beam tilt and obstruction by a moving object, there is asymmetric OAM spectrum broadening that depends on the direction of the object’s lateral motion along an axis, **v**, perpendicular to the plane containing **z** and **n**.

## Results

**Figure 1:**
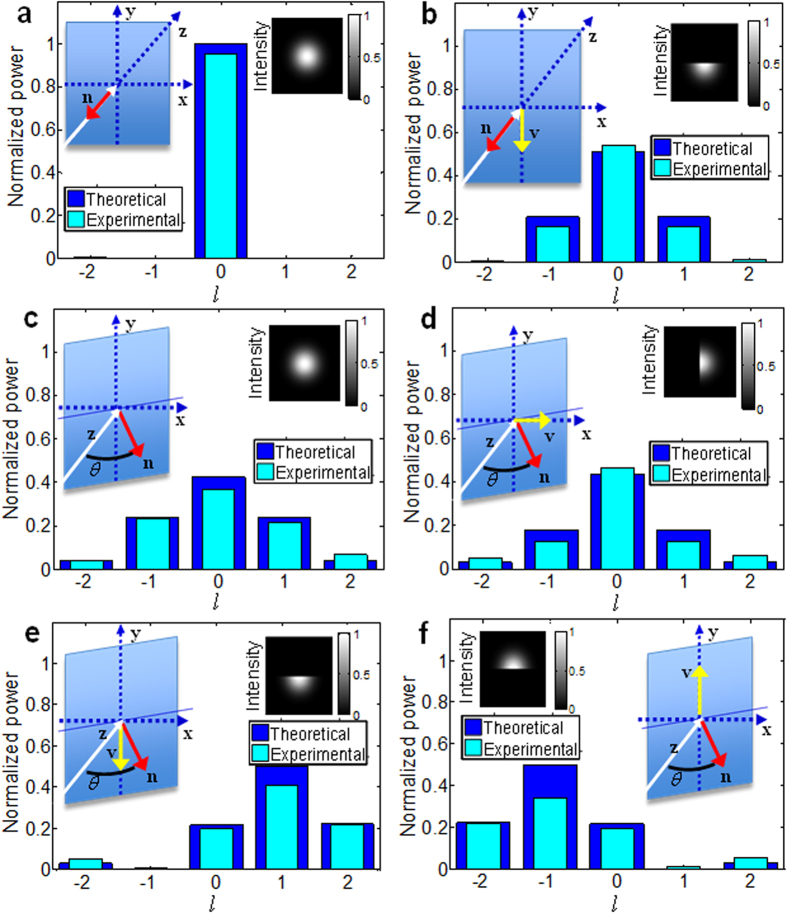
OAM power spectra and beam intensity profiles. (**a**) No tilt or obstruction. (**b**) No tilt, partial beam obstruction, **v** **=** **−y**. (**c**) Tilt *θ* = 5.28 × 10^−2^ ° between **n** and **z**. (**d**) Tilt *θ* = 5.28 × 10^−2^ ° between **n** and **z**, **v** **=** **x** coplanar with **n** and **z**. **(e)** Tilt *θ* = 5.28 × 10^−2^ ° between **n** and **z**, **v = −y** perpendicular to both **n** and **z**, asymmetric spectrum broadening about *l* = 0 favoring *l* = +1 over *l* = −1. (**f**) Tilt *θ* = 5.28 × 10^−2^ ° between **n** and **z**, **v = y** perpendicular to both **n** and **z**, asymmetric spectrum broadening about *l* = 0 favoring *l* = −1 over *l* = +1. The beam axis is **z** (white arrow), **n** (red arrow) is the measurement axis normal to the detector surface (blue parallelogram), and **v** (yellow arrow) is the lateral motion axis with the arrow indicating motion direction. Both **x** and **y** are orthogonal to both **z** and **n**. The beam center of gravity is defined to lie at **x = y** = 0 and follows a Gaussian beam intensity profile.

Figure 1 shows theoretical and experimental OAM power spectra for |*l* | ≤2 for a launched Gaussian beam. For a non-obstructed beam (Fig. 1a), we observe that if the beam and measurement axes are collinear (**z** || **n**), power is concentrated in the OAM launch state (*l* = 0). If the beam is partially obstructed (Fig. 1b) or if a tilt, *θ*, is imposed between the beam and measurement axes (Fig. 1c), symmetric broadening of the OAM spectrum occurs about *l* = 0. For simultaneous tilt and beam obstruction by an object moving along axis **v** = **x** that is coplanar with **z** and **n** (Fig. 1d), the spectrum broadening about *l* = 0 remains symmetric. However, if the lateral motion axis **v** is perpendicular to the plane containing **z** and **n**, we observe an OAM spectral asymmetry that corresponds to the lateral motion direction along **v**, as shown in Fig. 1e for **v** = **−y** and Fig. 1f for **v** = **y**. Moreover, through a simple rotation of the co-ordinate system of Fig. 1e,f, we find that lateral motion can be detected along an arbitrary axis **v** based on the same principles.

For small tilt angles in the paraxial approximation, the majority of power will reside in OAM states nearest to the launch state^21^. The asymmetry of OAM transmission in Fig. 1e,f can therefore be assessed by measuring the power difference between the two OAM states on either side of the launch state. For a Gaussian beam, this may be done by examining the ratio, *R*, between the power, *P*_*l*_ , in *l* = +1 and *l* = −1, where *R* is defined in units of decibels (dB) as


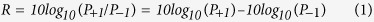


Referring back to Fig. 1e,f, we note that **v** **=** **−y** would result in *R* > 0 while **v** **=** **y** would yield *R* < 0, such that the sign of *R* can be used to determine the lateral motion direction of the remote object along an axis, **v**.

**Figure 2:**
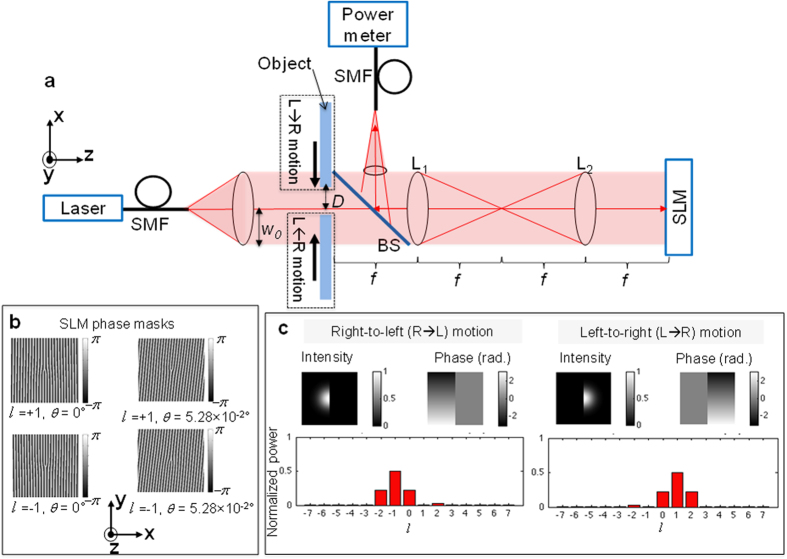
Experimental setup. (**a**) Gaussian beam collimation, launch, obstruction, and imaging onto a spatial light modulator (SLM). (**b**) SLM phase masks illustrating variable beam tilt control in the **y**–**z** plane via digital programming. (**c**) Power measurements for |*l* | ≤ 7 for left-to-right and right-to-left lateral motion of the object.

Figure 2 presents the experimental setup. A light beam from a 1550nm laser source is collimated to a waist size *w*_*0*_ ≈ 0.75 mm and obstructed along the **x-**axis by an object larger than the beam waist. The amount of beam obstruction is controlled by moving the object using a micrometer translation stage, and is measured in terms of distance, *D*, of the object from the beam center (Fig. 2a). A 4*f* system comprising lenses L_1_, L_2_ (focal length *f* = 20 cm) images the obstructed beam onto a 0–2*π* reflective liquid crystal on silicon spatial light modulator (SLM). A variable tilt in the *y–z* plane is imposed onto the SLM by digitally programming its spatial phase mask (Fig. 2b). The 4*f* system and a beam splitter (BS) image the obstructed and tilted beam onto a collimator lens, coupling it into a second SMF. The SMF is terminated by a power meter and the optical power in OAM states associated with |*l*| ≤7 is measured by displaying the corresponding spiral phase mask on the SLM and recording the power meter output (Fig. 2c). The power ratio, *R*, is computed according to equation (1) based on power measurements for *l* = +1 and *l* = −1. The experimental OAM spectra of Fig. 1 were obtained by modifying the setup of Fig. 2a,b such that the SLM tilt was imposed in the *x*–*z* plane and the object was translated along the **y**-axis. To emulate lateral motion of the object along an arbitrary axis, the SLM phase mask (Fig. 2b) was rotated clockwise in the *x–y* plane from 0 to 360°, and *R* was recorded for half-beam obstruction (*D* = 0) versus the phase mask rotation angle, first for an SLM tilt in the *x*–*z* plane and then for a tilt in the *y–z* plane.

**Figure 3:**
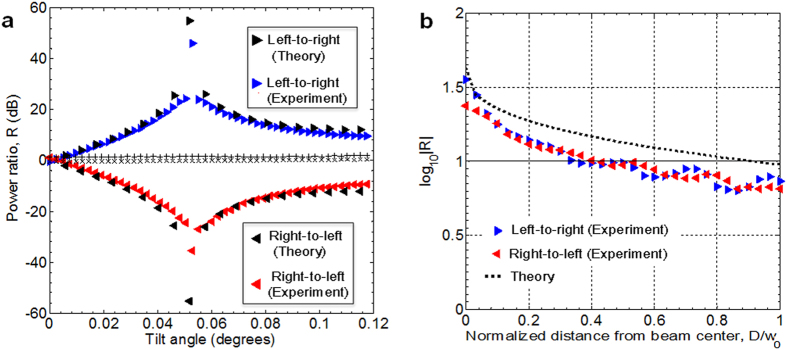
Experimental results. (**a**) Power ratio, *R*, versus tilt angle in the **y**–**z** plane for half-beam obstruction (*D* = 0) by an object moving along the **x**-axis. The sign of *R* discriminates the lateral motion direction. Experimental data are also shown for left-to-right (×) and right-to-left (+) motion versus tilt in the *x−z* plane. (**b**) The logarithm of |*R*| versus distance of the obstructing object from the beam center. The solid line corresponds to |*R*| = 10 dB.

**Figure 4:**
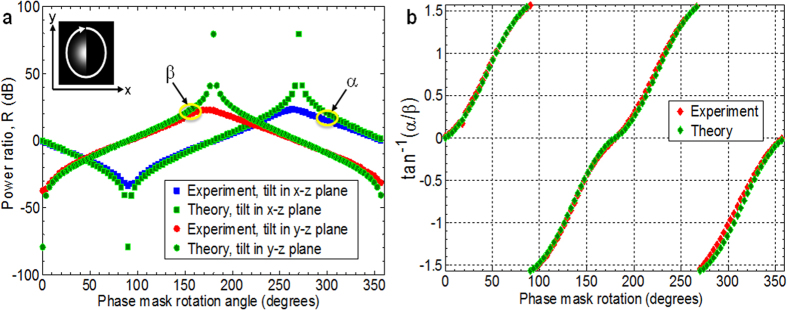
Experimental results. (**a**) Power ratio, *R*, for clockwise phase mask rotation (degrees) with *D* = 0 and tilt angle *θ* = 5.28 × 10^−2^ ° in the *x−z* plane (curves labelled α) and *y−z* plane (curves labelled β). The effective rotation of the obstruction is as shown in the inset. (**b**) Inverse tangent of α/β identifies arbitrary lateral motion axis orientation. The sign of *R* (Fig. 4a) removes angular ambiguity and discriminates lateral motion direction.

As shown in Fig. 3a, for tilt angles *θ* ≥ 10^−2^ ° in the *y–z* plane, the sign of *R* discriminates lateral motion along the **x**-axis, while the magnitude of *R* peaks at a tilt angle (in this case, 5.28 × 10^−2^) which generally depends on the beam’s waist size and wavelength. Effectively, the tilt angle corresponds to a phase gradient across the beam that arises from a superposition of intrinsic OAM states^18^. Figure 3b shows that for fixed tilt angle *θ* = 5.28 × 10^−2^ ° in the *y–z* plane, when the object is blocking one quarter to one half of the beam (0 ≤ *D*/*w*_*0*_ ≤ 0.25), |*R*| ≥10 dB for both left-to-right and right-to-left motion. The sign of *R*, however, is opposite for these two cases (Fig. 3a). A ≥ 20 dB power difference is thus observed for left-to-right versus right-to-left object motion over this beam obstruction range, such that a simple thresholding operation on *R* can be used to identify the motion direction. For clockwise phase mask rotation of the half-obstructed beam at tilt angle *θ* = 5.28 × 10^−2^ ° in the *x–z* and *y–z* planes (Fig. 4a), the α and β curves essentially track the sine and cosine of the phase mask rotation angle. The inverse tangent of α/β (Fig. 4b) can therefore be used to identify arbitrary lateral motion axis orientation. As shown in Fig. 4b, there is angular uncertainty^24^, which is disambiguated by the sign of *R* (Fig. 4a). We note that the presented method can measure an arbitrary lateral motion axis orientation along the x-axis and y-axis simultaneously by, for example, displaying a combination of masks on the SLM that are tilted in the y–z and x–z planes, each corresponding to different SMFs via different spatial frequencies.

We have experimentally shown that by analyzing the change in orbital angular momentum of a tilted light beam eclipsed by a moving object, lateral motion of the object can be detected in an arbitrary direction using a single light beam and without object image reconstruction. These findings extend OAM-based remote sensing to detection of non-rotational qualities of objects and may have applications in other electromagnetic wave regimes.

## Discussion

We note that the presented method can be extended to also determine lateral velocity by measuring the power ratio, *R*, as a function of time. Compared to lateral velocity measurement via laser Doppler velocimetry^25^, the OAM-based approach would have the advantages of using a single light beam and determining the presence of the remote object and direction of obstruction even when the obstructing object is not moving. Analogously to the use of Doppler to detect an object’s motion by mapping it to changes in light’s frequency, this method detects an object’s motion by mapping it to changes in light’s OAM. The presented method thus detects lateral motion in an arbitrary direction through a direct mapping between obstruction caused by remote object motion and extrinsic OAM of the light beam. The simple truncation denotes a reasonable regime where the object size is much greater than the size of the light beam. For certain applications, the presented approach may be more computationally efficient than camera-based machine vision systems. We also note that this method is related to modal decomposition of an image using a basis of OAM modes^26^, and that other mode bases could also potentially be used. Due to their inherent rotational symmetry, OAM modes have only been used for remote sensing of rotational qualities of objects^16^,^17^. In contrast, our work extends OAM-based remote sensing to detection of non-rotational qualities of objects.

With respect to camera-based object tracking, our method is analogous to the method of contour tracking that uses contour object representation with edge feature selection. Conventional computational algorithms used in contour tracking to identify edges include Canny edge detection and Sobel filters, which can require a minimum of nine camera pixels and can involve the application of a Gaussian filter, computation of an intensity gradient, non-maximum suppression, double thresholding, and hysteresis^27^. Comparatively, the OAM-based method uses the equivalent of four camera pixels (four SMFs connected to four photodiodes) and simply calculates the difference in powers between *l* = +1 and *l* = −1 OAM modes and then makes a decision based on the value of the ratio.

We moreover note that the configuration of vectors **z**, **n**, and **v** in Fig. 1e,f is geometrically analogous to the configuration of the so-called vector “chiral triad” for light transmission through a non-chiral metamaterial that exhibits what has been referred to as extrinsic chirality^28^,^29^. Extrinsic chirality can be understood in terms of a power difference between the right and left circular polarization states which arises from transmission through the metamaterial but is not ascribed to internal metamaterial structure^28^,^29^. Rather, it is attributed to an overall experimental configuration defined by three vectors— the normal to the surface of the metamaterial, a vector of asymmetry, and the light beam’s direction of propagation^29^. In Fig. 1e,f, the measurement axis **n** is the normal to the (detector) surface, the lateral motion axis **v** is the vector of asymmetry, and the direction of propagation of the light beam remains the **z** axis. The vector triads of Fig. 1e,f are chiral (mirror images that can’t be superimposed), and exhibit correspondence with a power difference between *l* = +1 and *l* = −1. We note that although we observe this geometric analogy, the underlying physical mechanisms of light-matter interaction for light’s circular polarization and light’s OAM are distinct^30^. We also note that although we have considered an obstruction that is equivalent to a simple truncation on one side of the light beam, it may be possible to extend this method to more complicated obstructions. It is likely that the resulting OAM spectra of more complicated obstructions will be more complex. Nonetheless, the approximation of an object as a simple truncation on one side of the light beam comprises a reasonable regime where the size of the object, such as a human hand or a vehicle, is much larger than the size of the light beam.

## Methods

We consider the complex and scalar amplitude of an arbitrary light beam given by 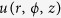 where 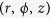 are cylindrical coordinates. The light beam can be described as a linear combination of OAM basis states^6^:


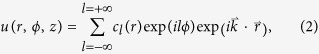


where *l* = 0, ±1, ±2, …, 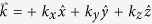 and 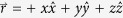, 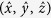 are Cartesian unit vectors, 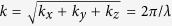, *λ* is the light beam’s wavelength, and 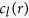 are the normalized complex coefficients of the OAM basis states given by:


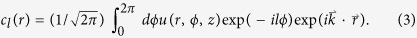


The power in each OAM basis state is computed as 
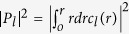
, and the compilation of values 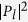 is the light beam’s OAM spectrum. The theoretical OAM spectrum of a beam truncated by an aperture (i.e. obstructed) can be calculated numerically^12^. The OAM spectra of Fig. 1 were numerically calculated using equations (2) and (3), wherein the amplitudes of a Gaussian beam when it is tilted and obstructed in various combinations are given next.

The amplitude of a Gaussian beam 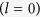 as shown in Fig. 1a, propagating along the 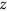-axis, is given by:


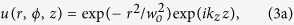


where 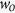 is the beam waist size.

The amplitude of a Gaussian beam when it is obstructed in the 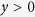 half-space of the 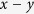 plane, as shown in Fig. 1b, is given by:


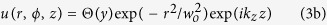


where 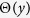 is the Heaviside step function.

The amplitude of a Gaussian beam when it is tilted with respect to the 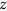-axis in the 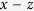 plane, as shown in Fig. 1c, is given by:


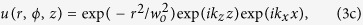


where 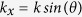, *θ* denoting the tilt angle. The amplitude of a Gaussian beam when it is tilted in the 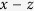 plane and obstructed in the 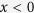 half-space of the 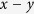 plane, as shown in Fig. 1d, is given by:


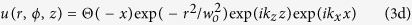


The amplitude of a Gaussian beam when it is tilted with respect to the 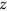-axis in the 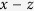 plane and obstructed in the 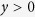 half-space of the 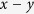 plane as shown in Fig. 1e is given by:


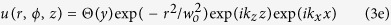


The amplitude of the Gaussian beam when it is tilted with respect to the 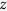-axis in the 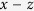 plane and obstructed in the 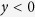 half-space of the 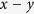 plane as shown in Fig. 1f is given by:


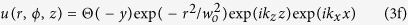


The amplitude of a Gaussian beam when it is obstructed in the 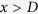 space, where 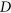 is the distance of the obstructing object from the beam center, is given by:


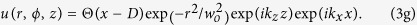


Using equation (2) together with equations (3f) and (3g), we numerically computed the ratio of the powers, *P*_*l*_, for 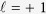 and 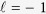 states of the OAM spectrum as a function of tilt angle, as shown in Fig. 3a for 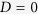. Moreover, for a fixed tilt angle, 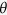, the ratio of the powers *P*_*l*_, for 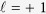 and 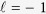 OAM states of the OAM spectrum can be numerically calculated as a function of *D*, as shown in Fig 3b.

## Additional Information

**How to cite this article**: Cvijetic, N. *et al*. Detecting Lateral Motion using Light's Orbital Angular Momentum. *Sci. Rep*. **5**, 15422; doi: 10.1038/srep15422 (2015).
